# A Case of Dens Fracture: A Pictorial Review and Discussion

**DOI:** 10.1155/2012/864160

**Published:** 2012-08-07

**Authors:** Bobby Desai, John Slish, Brandon Allen

**Affiliations:** Department of Emergency Medicine, University of Florida College of Medicine, 1329 SW 16th Street, P.O. Box 100186, Gainesville, FL 32610-0186, USA

## Abstract

Dens fractures are more common in the elderly and may present after minimal trauma and with minimal neck pain. The case reports a case of a significant fracture after minimal trauma, presenting with neurologic symptoms and minimal neck pain.

## 1. Introduction 

Dens fractures are the most common cervical spine fractures in persons above the age of 65 and account for approximately 5% to 15% of all cervical spine fractures [1–3]. We present a case of a patient with a significant fracture, yet minimal neck pain and some neurologic complaints. 

## 2. Case Presentation 

A 63-year-old female presented to our Emergency Department in transfer from an outside Emergency Department for neurosurgical evaluation of a diagnosed C2 fracture. The patient states that two weeks ago she had fallen asleep in her chair hitting the right side of her neck and shoulder. She immediately had minimal neck pain and was evaluated by her primary care physician. She had denied any new weakness or numbness though she reported chronic numbness in both of her upper extremities. She denied any bladder/bowel incontinence. At baseline, she had been ambulatory at home using a cane.

**Figure 1:**
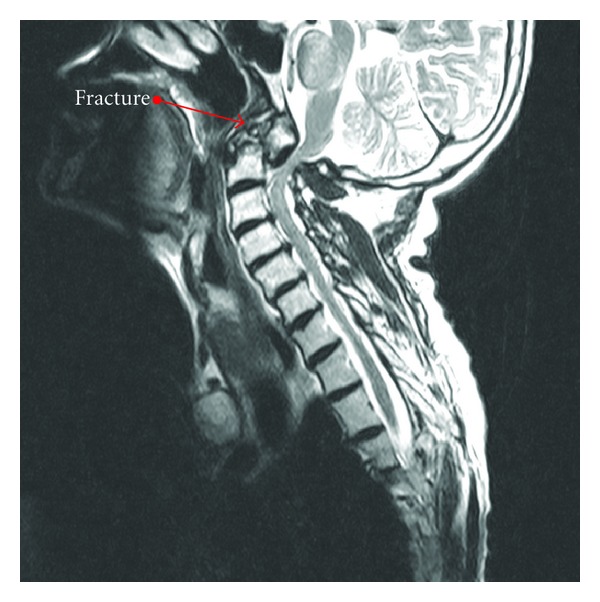
It shows a contrasted MRI with cervical spine fracture.

**Figure 2:**
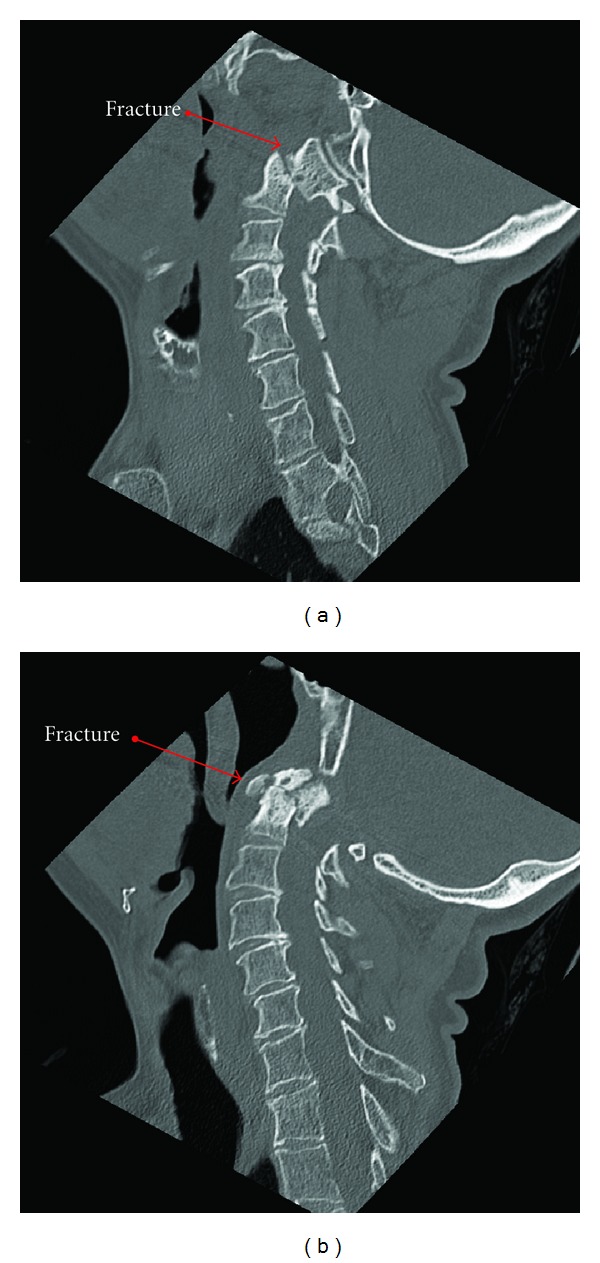
It shows noncontrasted MRI with cervical spine fracture.

**Figure 3:**
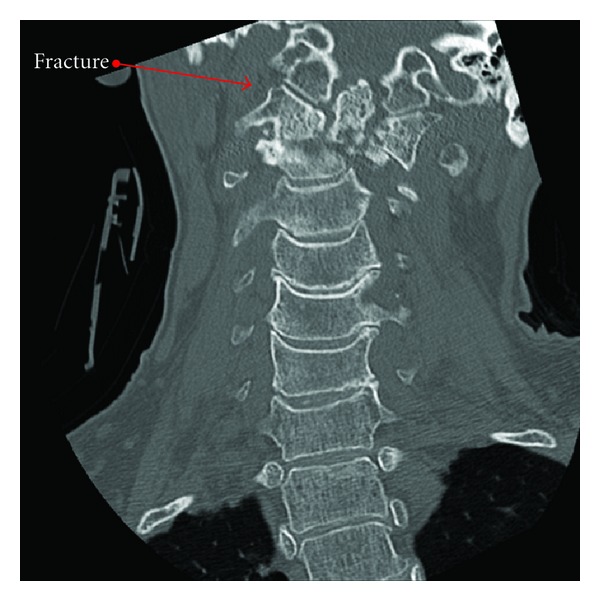
Frontal view.

An MRI performed at an outside institution revealed a type 2 C2 fracture (Figure 1) with spinal cord compression, and, due to this, the patient was subsequently transferred to our institution. Her physical exam revealed hyper-reflexia in the upper extremities bilaterally and a positive Hoffman's sign bilaterally. Otherwise, the neurologic exam was unrevealing. Computed tomography of the cervical spine was performed upon arrival for better delineation of the bony injury with confirmation of a C2 type 2 fracture with displacement (Figures 2(a), 2(b) and 3). Consequently, the patient was admitted to the neurosurgery service and underwent external immobilization with a halo vest for her injuries and did well with discharge home approximately one week after admission.

## 3. Discussion 

Dens fractures are classified as type I, II, or III. A type I fracture involves only the proximal tip of the odontoid process, while a type II fracture, like our patient's, passes through the base of the odontoid process. A type III fracture passes through the body of C2 [4]. 

Fractures of the dens can be seen at any time of life, but especially in young adolescents and also in those after 60. The etiology of these fractures is still controversial. Amling et al. attempted to examine this issue by removing the axis from autopsy cases for histomorphometric analysis [5]. They discovered that, due to intrinsic factors including a cortical thickness only 1/3 that of the axis, poor trabecular interconnection, and a decrease in its trabecular bone volume, the base of the dens is an area of increased weakness and thus fracture [5]. Due to these facts, they concluded that the obtained data suggest that the “bone structure of the axis is responsible for the location, the distribution and the frequency of fractures of the odontoid process” [5]. 

With aging come changes in the vertebral columns including decreased tensile, shear, and torsional strength. The strength of the spine depends on gender, age, and the rate of loading. In young patients, injuries usually occur with high rates of loading, whereas in the elderly, low loading rates in addition to decreased bone density may cause injury. This is a reasonable explanation to how such a trivial mechanism as striking your head while falling asleep in a chair would result in the described injury in contrast to the relatively high amount of force required to invoke the same injury pattern in a younger patient. 

Furthermore, with age comes a decrease in the range of motion. This occurs due to spondylosis and further degenerative changes in the supporting ligaments. Therefore, injury occurs near where the cervical spine is stiff and not pliant. This is most likely the cause of mobile upper cervical spine injuries in the elderly [6]. Due to a decrease in ligamental strength, hyperextension injuries are seen more frequently in the mid-to-lower cervical spine areas in the elderly [7]. The mechanism of injury in the elderly is typically hyperextension, whereas in the younger patient, hyperflexion injuries along with compression injuries are more common. 

Due to physiologic changes of aging, especially with perception of pain, diagnosis of cervical spine injuries may be difficult. For example, neck pain may be minimal, and the neurological component more significant including losing the ability to perform activities of daily living (ADL's). 

In regards to treatment strategies, treatment of type II fractures is controversial because of the high incidence of nonunion related to poor vascularity. Type II and III dens fractures are considered unstable and should be externally immobilized by a halo vest or fused surgically. Surgery often is undertaken for widely displaced fractures due to a poor chance of fusion and the known elevated risk of a nonunion/malunion and for those that fail external immobilization [4].
